# Capture method affects survival estimates and subsequent interpretation of ecological covariates for a long‐lived cervid

**DOI:** 10.1002/ece3.7494

**Published:** 2021-03-29

**Authors:** Katherine L. Brackel, Eric S. Michel, Bailey S. Gullikson, Jonathan A. Jenks, William F. Jensen

**Affiliations:** ^1^ Department of Natural Resource Management South Dakota State University USA; ^2^ Farmland Wildlife Populations and Research Group Minnesota Department of Natural Resources Madelia USA; ^3^ North Dakota Game and Fish Department Bismarck USA

**Keywords:** capture method, cause‐specific mortality, Odocoileus virginianus, survival, vaginal implant transmitters, white‐tailed deer neonates

## Abstract

Understanding what variables affect ungulate neonate survival is imperative to successful conservation and management of the species. Predation is commonly cited as a cause‐specific source of mortality, and ecological covariates often influence neonate survival. However, variation in survival estimates related to capture methodology has been documented with opportunistically captured neonates generally displaying greater survival than those captured via aid of vaginal implant transmitters (VITs), likely because of increased left truncation observed in the opportunistically captured datasets. Our goal was to assess whether 3‐ and 6‐month survival estimates varied by capture method while simultaneously assessing whether capture method affected model selection and interpretation of ecological covariates for white‐tailed deer neonates captured from three study sites from 2014 to 2015 in North Dakota and South Dakota, USA. We found survival varied by capture method for 3‐month neonate survival with opportunistically captured neonates displaying up to 26% greater survival than their counterparts captured via VITs; however, this relationship was not present for 6‐month survival. We also found model selection and subsequent interpretation of ecological covariates varied when analyzing datasets comprised of neonates captured via VITs, neonates captured opportunistically, and all neonates combined regardless of capture method. When interpreting results from our VIT‐only analysis for 3‐month survival, we found survival varied by three time intervals and was lowest in the first two weeks of life. Capture method did not affect 6‐month survival, which was most influenced by total precipitation occurring during 3 – 8 weeks of a neonate's life and percent canopy cover found at a neonate's capture site. Our results support previous research that capture method must be accounted for when deriving survival estimates for ungulate neonates as it can impact derived estimates and subsequent interpretation of results.

## Graphical abstract

Deriving survival estimates allows for a better understanding of what ecological variables affect offspring survival. However, we found that capture method affects both the derived survival estimate as well as model selection and ecological covariate interpretation for a long‐lived cervid neonate.

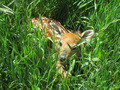

## 1 INTRODUCTION

Understanding the variability of vital rate parameters is important to better understand population dynamics for various species (Tuljapurkar & Caswell, 1997). Although variation in adult female survival generally has the greatest effect on population growth rates for ungulate populations, adult survival tends to be stable; conversely, offspring survival has less impact on population growth but is more variable (Chitwood et al., 2015; Gaillard et al., 1998, 2000; Raithel et al., 2007). Manipulating offspring survival to achieve various management and conservation goals is therefore a viable method to increasing population size (Coluccy et al., 2008; Crouse et al., 1987; Johnson et al., 2010). However, better understanding what specific variables affect offspring survival will likely increase effectiveness of management and conservation efforts.

Individual traits such as birth mass (Cook et al., 2004; Lomas & Bender, 2007; Shuman et al., 2017),capture age (Grovenburg et al., 2014), and birth date (Michel, Gullikson, et al., 2020; Plard et al., 2015), and environmental variables including weather (Ginnett & Young, 2000; Michel et al., 2018; Warbington et al., 2017), landscape composition and configuration (Gingery et al., 2018; Gulsby et al., 2017; Michel et al., 2018), and cohort effects (Douhard et al., 2013; Gaillard et al., 2003; Pigeon et al., 2017) affect ungulate offspring survival. Other factors such as maternal body condition may also affect offspring survival as ungulate mothers in better body condition are more likely to produce larger, healthier offspring with greater chances of survival than those in poor body condition (Carstensen et al., 2009; Duquette et al., 2015; Shallow et al., 2015). Additionally, predation is generally the largest natural cause of offspring mortality for several ungulate species (white‐tailed deer, Odocoileus virginianus, Chitwood et al., 2015; Grovenburg et al., 2011; elk, Cervus canadensis, Brodie et al., 2013; Griffin et al., 2011; moose, Alces alces, Keech et al., 2011, Severud et al., 2019; pronghorn, Antilocapra americana, Jacques et al., 2007, 2015); however, the number of predators an ungulate population is exposed to does not necessarily equate to increased mortality (Kautz et al., 2019). Consequently, factors affecting offspring survival can be area‐specific (Grovenburg et al., 2011); therefore, understanding which factors most influence offspring survival for a given ungulate population is warranted.

Although there are several ecological variables that affect ungulate offspring survival, field methodology can also affect derived survival estimates for a population. Derived survival estimates tend to be greater for opportunistically captured neonates compared with those captured via vaginal implant transmitters (VITs) due to increased left truncation in the opportunistically captured datasets (black‐tailed deer, *O. hemionus sitkensis*, Gilbert et al., 2014; white‐tailed deer, Chitwood et al., 2017; Dion et al., 2020). This variation can affect management and conservation efforts when survival estimates are needed to model population growth rates and abundance as these metrics are often used to determine the number of individuals that can be sustainably harvested from a population. Model selection and interpretation of the effects of ecological covariates on ungulate neonate survival can also vary by capture method (Gilbert et al., 2014). Therefore, assessing how capture method affects both derived survival estimates and interpretation of the relationship between ecological covariates and survival will further understanding of the population dynamics for a given species within an ecosystem.

**FIGURE 1:**
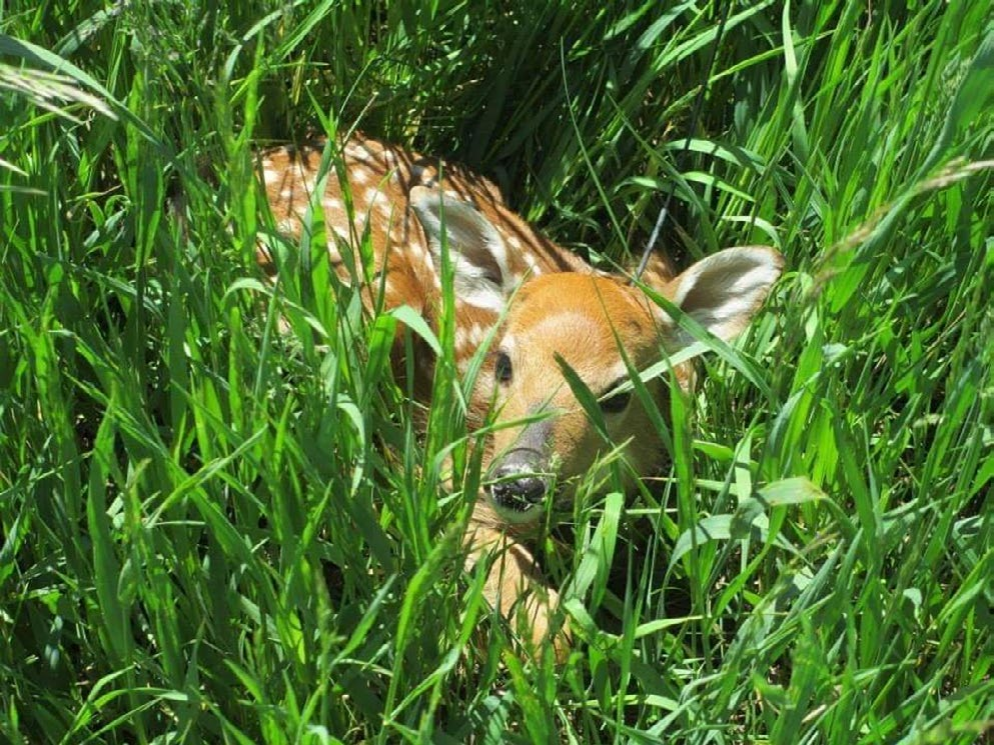
Derived survival estimates, model selection, and interpretation of ecological covariates for white‐tailed deer neonates (*Odocoileus virginianus*) may differ between capture methods

Our objective was to assess variation in survival estimates and subsequently assess potential variation in model selection and ecological covariate interpretation related to capture method for white‐tailed deer neonates (Figure 1) captured from three study areas in North Dakota and South Dakota, USA. We also assessed how individual traits (capture age, birth date, birth mass, sex) and environmental covariates (percent canopy cover, precipitation, distance to road, distance to water, cohort effects) affected neonate survival through 3 months (neonates) and 6 months (juveniles) of age. We predicted neonates captured via VITs would display decreased survival compared with opportunistically captured neonates because opportunistically captured neonates survive a particularly vulnerable period (e.g., the first few days of life; Chitwood et al., 2017; Dion et al., 2020; Gilbert et al., 2014). We also predicted survival would increase with increased birth mass (Cook et al., 2004; Lomas & Bender, 2007; Shuman et al., 2017), age (Grovenburg et al., 2011; Nelson & Woolf, 1987; Rohm et al., 2007), canopy cover (Rohm et al., 2007; Sternhagen, 2015), and increased distance from the nearest road (Rost & Bailey, 1979; Stankowich, 2008). We predicted that survival would decrease with delayed birth dates (Plard et al., 2015, Michel, Strickland, et al., 2020), increased precipitation (Dion et al., 2020); Warbington et al., 2017, and increased distance from water (Adams & Hayes, 2008; Ditchkoff, 2011; Long et al., 2009). Finally, we predicted survival would be lower for females than for males (Shuman et al., 2017).

## 2 MATERIALS AND METHODS

### 2.1 Study area

**FIGURE 2:**
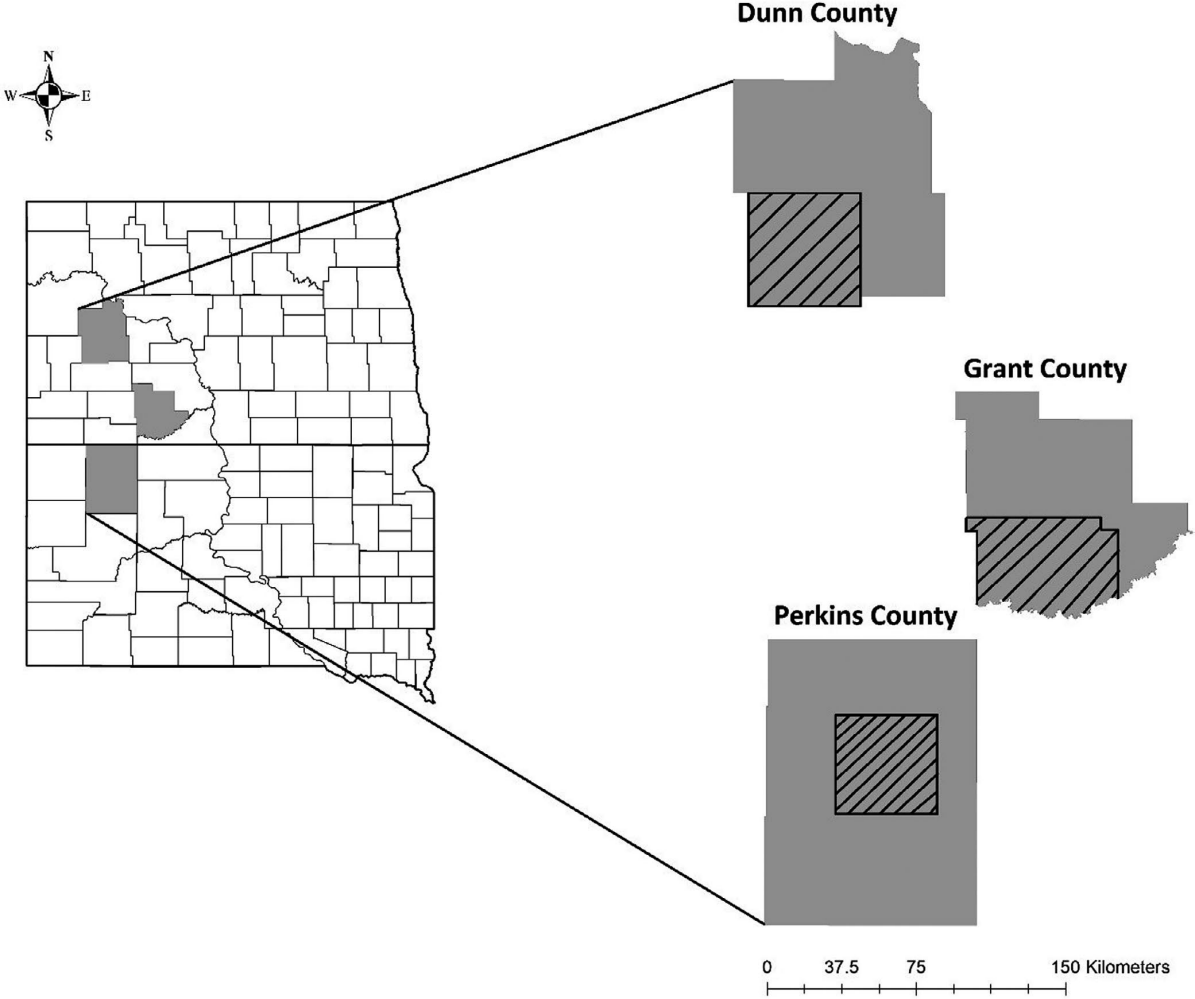
Study areas where we captured and radio‐collared adult female and neonatal white‐tailed deer in Dunn and Grant counties, North Dakota, and Perkins County, South Dakota, USA. Dashed lines indicate deer capture areas within each study area

We investigated white‐tailed deer neonate cause‐specific mortality and survival in Grant and Dunn counties, North Dakota, and Perkins County, South Dakota (Figure 2) during 2014–2015. All counties were located in the Northwestern Great Plains Level III Ecoregion (Bryce et al., 1998). We focused deer capture in a 1492‐km^2^ area in the southwestern portion of Dunn County, an 1865‐km^2^ area in the southwestern portion of Grant County, and a 1492‐km^2^ area in the central portion of Perkins County. Grasslands, cropland, and forested areas were the most common cover types and ranged from 60% to 86%, 11 to 26%, and 0.01 to 9%, respectively (Cropland Data Layer, U. S. Department of Agriculture, 2015). Thirty‐year mean annual precipitation ranged from 41.2 cm (Grant County) to 44.9 cm (Perkins County), and variation in thirty‐year mean monthly temperature was greatest in Perkins County ranging from −12.1°C to 30.3°C (North Dakota State Climate Office, 2016).

Neonates surviving to 6 months were available for harvest. Recreational hunting season dates were similar across study areas and occurred from 29 August 2014 to 4 January 2015 and 4 September 2015 to 3 January 2016 in North Dakota. Recreational hunting occurred from 27 September 2014 to 15 January 2015 and from 26 September 2015 to 15 January 2016 in South Dakota.

### 2.2 Capture and monitoring

We captured adult female (≥ 1.5‐year‐old) white‐tailed deer via helicopter net guns (Native Range Capture Service, Elko, NV, USA). We then affixed very high frequency (VHF) radio‐collars (model M2610B; Advanced Telemetry Systems, Inc., Isanti, MN) to individuals and inserted vaginal implant transmitters (103 females; Advanced Telemetry Systems, Inc., Isanti, MN, USA) to aid in neonate capture (Bowman & Jacobson, 1998; Carstensen et al., 2003; Swanson et al., 2008).

We captured white‐tailed deer neonates from 23 May to 23 June in Dunn and Grant Counties, in North Dakota, and in Perkins County, South Dakota, during 2014–2015. We monitored VITs daily and subsequently searched for neonates near expelled VITs. We also searched for neonates in areas of known parturition habitat and near females who showed postpartum behavior such as isolation or fleeing short distances when approached (Grovenburg et al., 2010; Rohm et al., 2007). We then captured neonates by hand or net once we located them. We restrained and blindfolded neonates upon capture, determined sex, recorded body mass (kg), and fitted individuals with a M4210 expandable breakaway radio‐collar (Advanced Telemetry Systems, Isanti, MN, USA). We wore sterile rubber gloves, used no‐scent spray, stored radio‐collars and other equipment in natural vegetation, and kept handling time under five minutes when possible to reduce capture‐related mortality. We only determined sex of radio‐collared neonates if individuals were wet from rain or afterbirth or were too large to fit in the weighing bag. All handling methods followed the American Society of Mammalogists guidelines for mammal care and use (Sikes et al., 2016) and were approved by the South Dakota State University Institutional Animal Care and Use Committee (Approval No. 13‐091A).

### 2.3 Statistical analysis

We monitored neonates daily for mortality during the first 30 days using aerial telemetry, omnidirectional whip antennas, and handheld telemetry equipment and then monitored them 2–3 times per week thereafter. We investigated mortalities immediately after detecting a mortality signal and transported carcasses to the North Dakota Game and Fish Department Wildlife Health Laboratory in Bismarck, North Dakota, USA, to confirm proximate cause of death.

We summarized weekly neonate mortality from telemetry data (Grovenburg et al., 2014). We estimated 3‐month (capture to 12 weeks) survival rates using the Kaplan–Meier method (Kaplan & Meier, 1958) for nonstaggered entry and 6‐month (capture to 24 weeks) survival rates using staggered entry (Pollock et al., 1989) via known fate models in Program MARK version 6.0 (Cooch & White, 2016; White & Burnham, 1999). We used a nonstaggered entry design for 3‐month survival because we investigated how survival varied by age interval for 3‐month survival only (Grovenburg et al., 2014). We considered models within 2 ∆AICc of the top model as competing (Burnham & Anderson, 2002).

We developed up to 21 models to describe factors that most affected neonate survival (S) at 3 and 6 months of age. Predictor variables included year (to assess cohort effects), age interval 1 (2‐stage age interval: 0–2 weeks, 3 + weeks), age interval 2 (3‐stage age interval: 0–2 weeks, 3–8 weeks, 9 + weeks), capture type (VIT or opportunistic), distance from capture site to nearest road (km) and distance from capture site to nearest water body (stream or stock pond, km), percent canopy cover at neonate capture site, precipitation during age intervals (0–2 weeks, 3–8 weeks, 9–12 weeks, 13–24 weeks), sex, birth date, birth mass, and capture age (Table 1). We used hoof measurements to estimate capture age for all neonates (Haugen & Speake, 1958; Brinkman et al., 2004). We back‐calculated birth mass for individuals estimated to be > 1 day old using estimated age and estimated neonate mass gain of 0.215 kg per day (Verme, 1963). We assigned mean mass (*n* = 27) and mean hoof measurements (*n* = 33) of neonates captured within the same week to neonates that were too wet or too large to weigh or take hoof measurements (i.e., only sex was obtained, and a radio‐collar was placed on the individual). Finally, we assigned capture mass as birth mass for individuals estimated to be ≤ 1 day old. We refer to all body mass measurements obtained at capture and estimated body mass for neonates as body mass. We assessed whether capture method affected derived survival estimates, model selection, and our subsequent interpretation of ecological covariates by including capture method in our candidate model set for all neonates. We then excluded capture method from our candidate set and further assessed how model selection and interpretation varied by analyzing a dataset that included neonates captured via VITs, neonates opportunistically captured, and all neonates combined regardless of capture method. Finally, we compared birth mass and capture age between neonates captured from VITs and neonates captured opportunistically using analysis of variance (ANOVA) in Program R (R Core Team, 2016 version 3.3.1; Themeau, 2015). We considered all variables important if their 95% confidence intervals (95% CI) excluded zero.

**TABLE 1:** Definitions of parameters used in 3‐month and 6‐month model sets to assess fawn survival in Dunn and Grant counties, North Dakota, and Perkins County, South Dakota

Models	Description
Year	Survival varied by capture year of fawn
Age	Survival varied by age (days) at capture of fawn
Age + Sex	Survival varied by age (days) at capture and sex of fawn
Age + Mass	Survival varied by age (days) at capture and mass (kg) of fawn at birth
Age + Mass +Sex	Survival varied by age (days) at capture, mass (kg) at birth, and sex of fawn
Birth Date	Survival varied by birth date (Julian date) of fawn
^a^ Int 1	Survival varied by age of fawn in 2 intervals
^b^Int 2	Survival varied by age of fawn in 3 intervals
Mass	Survival varied by mass (kg) at birth of fawn
Mass + Sex	Survival varied by mass (kg) at birth and sex of fawn
Sex	Survival varied by sex of fawn
Capture	Survival varied by capture type
Canopy	Survival varied by canopy cover (%)
Canopy + Precip 1	Survival varied by canopy cover (%) and precipitation during 0–2 weeks of fawn life
Canopy + Precip 2	Survival varied by canopy cover (%) and precipitation during 3–8 weeks of fawn life
Canopy + Precip 3	Survival varied by canopy cover (%) and precipitation during 9–12 weeks of fawn life
Canopy + Precip 4	Survival varied by canopy cover (%) and precipitation during 13–24 weeks of fawn life
Road	Survival varied by distance to nearest road (km)
Water	Survival varied by distance to nearest water source (km)
Changing Survival (t)	Survival varied weekly across time period
Constant Survival (.)	Survival remained constant across time period

^a^ 2‐stage age interval: 0–2 weeks and 3 + weeks

^b^ 3‐stage age interval: 0–2 weeks, 3–8 weeks, and 8 + weeks

## 3 RESULTS

We captured and radio‐collared 84 neonates (51 males, 33 females) from 23 May to 20 June 2014 and 73 neonates (44 males, 28 females, 1 unknown) from 27 May to 23 June 2015 totaling 157 radio‐collared neonates. Adult females retained 76 of 103 (74%) VITs until parturition. We observed 2 capture‐related mortalities during 2015 and removed those individuals from all analyses. Of the 155 neonates available for analysis, we captured 97 opportunistically and captured 58 via aid of VITs. Mean capture age was 4.1 ± 3.7 days (*n* = 155), and mean capture mass was 2.9 ± 1.0 kg (*n* = 155) for all neonates.

We documented 34 (22%) neonate mortalities between capture and 3 months of age and 44 (28%) neonate mortalities between capture and 6 months of age (Table 2). We documented 28 (64%) male and 16 (36%) female mortalities across study areas and years. Predation was the primary source of natural mortality (61%), whereas hunter harvest (9%) was the only source of human‐related mortality (Table 2). We identified 21 mortalities from neonates captured from VITs compared with 26 mortalities from neonates captured opportunistically. Finally, we documented 2 stillbirths from mothers who were equipped with VITs, whereas we did not document any stillbirths associated with mothers who did not have VITs inserted.

**TABLE 2:** Cause‐specific mortality for radio‐collared white‐tailed deer neonates up to 6 months of age in Dunn and Grant counties, North Dakota, and Perkins County, South Dakota, USA, during 2014–2015

	Year		
Mortality Cause	2014	2015	Total
Predation	10	6	16
Suspected Predation	9	2	11
Disease	4	0	4
Abandoned	1	1	2
Harvest	2	2	4
Unknown	4	3	7
Total	30	14	44

### 3.1 Survival Model Comparisons

Our first model set assessed whether capture type affected 3‐month survival. Our best 3‐month survival model was S(Year), which accounted for a moderate amount of model weight (*w_i_* = 0.44; Table 3). Overall survival was 0.78 (95% CI = 0.702 – 0.847) and differed between years (β = −1.188, 95% CI = −1.987 – −0.389). Survival was greater in 2015 (S = 0.88, 95% CI = 0.776 – 0.938) than in 2014 (S = 0.66, 95% CI = 0.547 – 0.755). S(Capture) was, however, a competing model but accounted for low model weight (ΔAIC_c_ = 1.15, *w_i_* = 0.25; Table 3). Nevertheless, this model indicated that capture type affected survival (β = −1.040, 95% CI = −1.731 ‐ −0.348). Overall survival was 0.78 (95% CI = 0.700 – 0.842) with neonates captured via VITs displaying decreased survival (S = 0.62, 95% CI = 0.487 – 0.740) compared with neonates captured opportunistically (S = 0.84, 95% CI = 0.753 – 0.905). Neonates captured using VITs were younger ($\overset{‐}{x}$ = 1.8 ± 2.7 days, *n* = 58) than opportunistically captured neonates ($\overset{‐}{x}$= 6. 0 ± 3.1 days, *n* = 97; *F_1,153_* = 71.170, *p* <.001). Estimated birth mass for neonates captured using VITs was greater ($\overset{‐}{x}$= 3.2 ± 0.9 kg, *n* = 58) than estimated birth mass for opportunistically captured neonates ($\overset{‐}{x}$= 2.8 ± 1.0, *n* = 97; *F_1,153_* = 4.529, *p* =.035).

**TABLE 3:** A *priori* models used to estimate 3‐month survival for 155 radio‐collared white‐tailed deer neonates in Dunn and Grant counties, North Dakota, and Perkins County, South Dakota, USA, during 2014–2015. Models within 2 ΔAIC_c_ are competing, *w_i_* indicates model weight, and *K* indicates number of parameters calculated within a model

Model	ΔAIC_c_	*w_i_*	*K*	Model likelihood
S(Year)	0.00	0.44	2	1.00
S(Capture)	1.15	0.25	2	0.56
^a^S(Canopy + Precip2)	3.09	0.09	3	0.21
S(Age)	4.45	0.05	2	0.11
S(Canopy)	4.98	0.04	2	0.08
^b^S(Int2)	5.74	0.02	3	0.06
^a^S(Canopy + Precip3)	6.04	0.02	3	0.05
S(Age + Sex)	6.36	0.02	3	0.04
S(Age + Mass)	6.45	0.02	3	0.04
^a^S(Canopy + Precip1)	6.99	0.01	3	0.03
S(.)	7.98	0.01	1	0.02
S(Age + Mass+Sex)	8.36	0.01	4	0.02
S(Birth Date)	8.50	0.01	2	0.01
S(Mass)	8.55	0.01	2	0.01
^c^S(Int1)	9.18	0.00	2	0.01
S(Road)	9.83	0.00	2	0.01
S(Sex)	9.97	0.00	2	0.01
S(Water)	9.99	0.00	2	0.01
S(Mass + Sex)	10.53	0.00	3	0.01
S(t)	45.54	0.00	17	0.00

^a^ Precipitation intervals (1:0–2 weeks; 2:3–8 weeks; 3:9–12 weeks; 4:13–24 weeks)

^b^ 3‐stage age interval: 0–2 weeks, 3–8 weeks, and 8 + weeks

^c^ 2‐stage age interval: 0–2 weeks and 3 + weeks

Given S(Capture) was a competing model, we excluded it from our candidate set of models and further assessed 3‐month survival for neonates captured via VITs only. S(Int2) was our best model and described 3‐month survival varying by three time intervals (0 – 2 weeks, 3 – 8 weeks, and 9 + weeks) but carried a low model weight (*w_i_* = 0.33; Table 4) with overall survival being 0.64 (95% CI = 0.507 – 0.755). Time interval had a positive effect on survival with survival generally increasing with time (0 – 2 weeks, S = 0.46, 95% CI = 0.200 – 0.693, β = 2.708, 95% CI = 0.1.943 – 3.473; 3 – 8 weeks, S = 0.56, 95% CI = 0.361 – 0.720, β = 3.004, 95% CI = 2.424 – 3.584; 9 + weeks, S = 0.92, 95% CI = 0.568 – 0.989, β = 4.997, 95% CI = 3.031 – 6.964). S(Canopy + Precip1) was competing but also carried a low amount of model weight (ΔAIC_c_ = 0.90; *w_i_* = 0.21). Overall survival for S(Canopy + Precip1) was 0.21 (95% CI = 0.044 – 0.618); however, there was only a weak relationship between percent canopy cover and survival (β = 0.021, 95% CI = −0.002 – 0.044), while total precipitation from 0 to 2 weeks of a neonate's life did not affect 3‐month survival (β = 0.465, 95% CI = −0.012 – 0.942). Therefore, we considered total precipitation from 0 to 2 weeks of life as an uninformative parameter (Arnold, 2010). All other models were ≥ 2.87 ΔAIC_c_ away from our best model.

**TABLE 4:** A *priori* models excluding capture method used to estimate 3‐month survival for 58 radio‐collared white‐tailed deer neonates captured via VITs in Dunn and Grant counties, North Dakota, and Perkins County, South Dakota, USA, during 2014–2015. Models within 2 ΔAIC_c_ are competing, *w_i_* indicates model weight, and *K* indicates number of parameters calculated within a model

Model	ΔAIC_c_	*w_i_*	*K*	Model likelihood
^a^S(Int2)	0.00	0.33	3	1.00
^b^S(Canopy + Precip1)	0.90	0.21	3	0.64
S(Canopy)	2.87	0.08	2	0.24
^b^S(Canopy + Precip3)	3.91	0.05	3	0.14
S(.)	4.06	0.04	1	0.13
^c^S(Int1)	4.15	0.04	2	0.13
^b^S(Canopy + Precip2)	4.25	0.04	3	0.12
S(Age)	4.74	0.03	2	0.09
S(Birth Date)	4.90	0.03	2	0.09
S(Water)	4.99	0.03	2	0.08
S(Sex)	5.01	0.03	2	0.08
S(Age + Sex)	5.24	0.02	3	0.07
S(Year)	5.85	0.02	2	0.05
S(Mass)	5.87	0.02	2	0.05
S(Road)	6.01	0.02	2	0.05
S(Mass + Sex)	6.35	0.01	3	0.04
S(Age + Mass)	6.54	0.01	3	0.04
S(Age + Mass+Sex)	7.26	0.01	4	0.03
S(t)	378.88	0.00	17	0.00

^a^ 3‐stage age interval: 0–2 weeks, 3–8 weeks, and 8 + weeks

^b^ Precipitation intervals (1:0–2 weeks; 2:3–8 weeks; 3:9–12 weeks; and 4:13–24 weeks)

^c^ 2‐stage age interval: 0–2 weeks and 3 + weeks

Our best model describing 3‐month survival for opportunistically captured neonates after excluding capture method from our candidate set was S(Canopy + Precip1). S(Canopy + Precip1) carried a low amount of model weight (*w_i_* = 0.24; Table 5) with overall survival being 0.90 (95% CI = 0.693 – 0.973). Percent canopy cover (β = 0.035, 95% CI = −0.013 – 0.082) and total precipitation from 0 to 2 weeks (β = −0.400, 95% CI = −0.906 – 0.105) did not affect neonate survival. S(Canopy) was a competing model and carried a low amount of model weight (ΔAIC_c_ = 0.44, *w_i_* = 0.20) with an overall survival of 0.78 (95% CI = 0.655 – 0.872). Percent canopy cover displayed a weak but positive relationship with 3‐month fawn survival (β = 0.041, 95% CI = −0.007 – 0.088). S(Canopy + Precip2) was also a competing model but again carried a low amount of model weight (ΔAIC_c_ = 0.57, *w_i_* = 0.18). Percent canopy cover displayed a positive but weak relationship with 3‐month neonate survival (β = 0.039, 95% CI = −0.008 – 0.085) while there was no relationship between total precipitation from 3 to 8 weeks and neonate survival (β = −0.342, 95% CI = −0.814 – 0.130). All other models were ≥ 2.45 ΔAIC_c_ from our best model.

**TABLE 5:** A *priori* models excluding capture method used to estimate 3‐month survival for 97 radio‐collared white‐tailed deer neonates captured opportunistically in Dunn and Grant counties, North Dakota, and Perkins County, South Dakota, USA, during 2014–2015. Models within 2 ΔAIC_c_ are competing, *w_i_* indicates model weight, and *K* indicates number of parameters calculated within a model

Model	ΔAIC_c_	*w_i_*	*K*	Model likelihood
^a^S(Canopy + Precip1)	0.00	0.24	3	1.00
S(Canopy)	0.44	0.20	2	0.80
^a^S(Canopy + Precip2)	0.57	0.18	3	0.75
^a^S(Canopy + Precip3)	2.45	0.07	3	0.29
S(Year)	2.65	0.06	2	0.27
S(.)	3.53	0.04	1	0.17
S(Sex)	4.10	0.03	2	0.13
S(Water)	4.64	0.02	2	0.10
S(Birth Date)	4.76	0.02	2	0.09
S(Mass)	5.05	0.02	2	0.08
S(Road)	5.19	0.02	2	0.07
^b^S(Int1)	5.29	0.02	2	0.07
S(Mass + Sex)	5.41	0.02	3	0.07
S(Age)	5.53	0.02	2	0.06
S(Age + Sex)	6.11	0.01	3	0.05
^c^S(Int2)	6.52	0.01	3	0.04
S(Age + Mass)	6.96	0.01	3	0.03
S(Age + Mass+Sex)	7.11	0.01	4	0.03
S(t)	43.36	0.00	11	0.00

^a^ Precipitation intervals (1:0–2 weeks; 2:3–8 weeks; 3:9–12 weeks; and 4:13–24 weeks)

^b^ 2‐stage age interval: 0–2 weeks and 3 + weeks

^c^ 3‐stage age interval: 0–2 weeks, 3–8 weeks, and 8 + weeks

After excluding capture method from the candidate set of models and further assessing survival for all neonates combined, S(Year) was our top model and accounted for a moderate amount of model weight (*w_i_* = 0.58; Table 6), indicating survival varied by year (β = −1.188, 95% CI = −1.987 ‐ −0.389). Overall survival was 0.78 (95% CI = 0.702 – 0.847) and was greater in 2015 (S = 0.88, 95% CI = 0.776 – 0.938) than in 2014 (S = 0.65, 95% CI = 0.547 – 0.755). All other models were ≥ 3.09 ΔAIC_c_ from our best model.

**TABLE 6:** A *priori* models excluding capture method used to estimate three‐month survival for 155 radio‐collared white‐tailed deer neonates regardless of capture type in Dunn and Grant counties, North Dakota, and Perkins County, South Dakota, USA, during 2014–2015. Models within 2 ΔAIC_c_ are competing, *w_i_* indicates model weight, and *K* indicates number of parameters calculated within a model

Model	ΔAICc	*w_i_*	*K*	Model likelihood
S(Year)	0.00	0.58	2	1.00
^a^S(Canopy + Precip2)	3.09	0.12	3	0.21
S(Age)	4.45	0.06	2	0.11
S(Canopy)	4.98	0.05	2	0.08
^b^S(Int2)	5.74	0.03	3	0.06
^a^S(Canopy + Precip3)	6.04	0.03	3	0.05
S(Age + Sex)	6.36	0.02	3	0.04
S(Age + Mass)	6.45	0.02	3	0.04
^a^S(Canopy + Precip1)	6.99	0.02	3	0.03
S(.)	7.98	0.01	1	0.02
S(Age + Mass+Sex)	8.36	0.01	4	0.02
S(Birth Date)	8.50	0.01	2	0.01
S(Mass)	8.55	0.01	2	0.01
^c^S(Int1)	9.18	0.01	2	0.01
S(Road)	9.83	0.00	2	0.01
S(Sex)	9.97	0.00	2	0.01
S(Water)	9.99	0.00	2	0.01
S(Mass + Sex)	10.53	0.00	3	0.01
S(t)	215.92	0.00	17	0.00

^a^ Precipitation intervals (1:0–2 weeks; 2:3–8 weeks; 3:9–12 weeks; and 4:13–24 weeks)

^b^ 3‐stage age interval: 0–2 weeks, 3–8 weeks, and 8 + weeks

^c^ 2‐stage age interval: 0–2 weeks and 3 + weeks

S(Canopy + Precip2) was our top model affecting 6‐month survival and accounted for a moderate amount of model weight (*w_i_* = 0.51; Table 7). Overall survival was 0.68 (95% CI = 0.587 – 0.759) with precipitation during 3 to 8 weeks negatively influencing juvenile survival (β = −0.461, 95% CI = −0.781– −0.142); however, there was only a moderate relationship suggesting canopy cover positively affected 6‐month survival (β = 0.016, 95% CI = 0.000 – 0.033). Mean precipitation from 3 to 8 weeks for surviving juveniles was 2.9 ± 0.8 cm (*n* = 84) compared with 3.3 ± 0.9 cm (*n* = 41) for juveniles that perished. Mean percent canopy cover at capture sites for surviving juveniles was ~ 20 ± 25% (*n* = 84) compared with ~ 11 ± 20% (*n* = 41) for juveniles that perished. S(Year) was a competing model and accounted for a low amount of model weight (ΔAIC_c_ = 1.01, *w_i_* = 0.31). Overall survival was 0.68 (95% CI = 0.588 – 0.761) and varied by year (β = −1.019, 95% = −1.714 ‐ −0.324). Survival was greater in 2015 (S = 0.80, 95% CI = 0.676 – 0.886) than in 2014 (S = 0.54, 95% CI = 0.424 – 0.657). All other models were ≥ 5.05 ∆AIC_c_ from our best model in our 6‐month survival model set. Given capture method was not a top model nor was it competing, we did not further assess how model selection, survival, and ecological covariate effects varied among analyses including those captured via VITs, those captured opportunistically, and all juveniles combined regardless of capture method.

**TABLE 7:** A *priori* models used to estimate six‐month survival of 155 radio‐collared white‐tailed deer fawns in Dunn and Grant counties, North Dakota, and Perkins County, South Dakota, USA, during 2014–2015

Model	ΔAIC_c_	*w_i_*	*K*	Model likelihood
^a^S(Canopy + Precip2)	0.00	0.51	3	1.00
S(Year)	1.01	0.31	2	0.60
S(Capture)	5.05	0.04	2	0.08
S(Canopy)	5.55	0.03	2	0.06
S(Age)	7.04	0.02	2	0.03
S(Road)	7.28	0.01	2	0.03
^a^S(Canopy + Precip4)	7.29	0.01	3	0.03
^a^S(Canopy + Precip3)	7.37	0.01	3	0.03
^a^S(Canopy + Precip1)	7.55	0.01	3	0.02
S(Birth Date)	8.09	0.01	2	0.02
S(.)	8.33	0.01	1	0.02
S(Mass)	8.96	0.01	2	0.01
S(Age + Mass)	9.02	0.01	3	0.01
S(Age + Sex)	9.02	0.01	3	0.01
S(Water)	10.33	0.00	2	0.01
S(Sex)	10.34	0.00	2	0.01
S(Mass + Sex)	10.97	0.00	3	0.00
S(Age + Mass+Sex)	11.00	0.00	4	0.00
S(t)	50.99	0.00	80	0.00

^a^ Precipitation intervals (1:0–2 weeks; 2:3–8 weeks; 3:9–12 weeks; and 4:13–24 weeks)

## 4 DISCUSSION

Our top model for 3‐month neonate survival was S(Year) indicating that environmental factors likely induced a cohort effect for neonates in our study. This supports other research where environmental factors experienced at birth affected survival, reproductive success, and individual phenotype (Douhard et al., 2013; Gaillard et al., 2003; Pigeon et al., 2017). Interestingly, S(Year) was no longer competing when we assessed models for neonates captured via VITs only nor for those neonates only captured opportunistically. Yet, S(Capture) was a competing model, which supported our prediction that survival would vary by capture method as neonates captured via VITs displayed up to a 26% lower survival rate than opportunistically captured neonates. This result also supports previous studies assessing variation in survival rates related to capture methods where survival estimates were 7 to 25% lower for ungulate neonates captured via VITs compared with opportunistically captured ungulate neonates (Chitwood et al., 2017; Dion et al., 2020; Gilbert et al., 2014). We found opportunistically caught neonates were about 6 days old, which supports Dion et al., 2020; (6‐days); however, variation in age between capture methods may be as little as 3.5 days (Kautz et al., 2019). Gilbert et al. (2014) simulated left truncation by removing black‐tailed deer neonates that died within 2 days of age from a known‐age dataset, which still resulted in increased survival estimates compared with survival estimates derived from datasets that did not contain left truncation data (i.e., VIT‐only data). Although variation in survival estimates related to the inability to capture neonates within the first seven days of life is intuitive (Chitwood et al., 2017; Dion et al., 2020), survival rates can vary even when failing to capture neonates < 2 days old (Gilbert et al., 2014). Alternatively, inserting a VIT is an intrusive method and we cannot discount the potential that the method may lead to birth complications and subsequently increased mortality at birth or during the initial days of life; though, the lack of stillbirths we found that were associated with adult females with a VIT does not support this potential result. Regardless, research designed to derive neonatal survival estimates should capture neonates via VITs or should acknowledge that derived survival estimates could be up to 26% lower than those derived from datasets focused on opportunistically captured neonates.

In addition to reporting variation in survival estimates related to capture method, Gilbert et al. (2014) reported variation in model selection and interpretation of ecological covariates related to grouping and subsequently analyzing neonate survival by capture method. Our results further support Gilbert et al. (2014) as we derived three different top models based on how we grouped and analyzed our data. For example, S(Int2) was our top model when only using neonates captured via VITs, S(Canopy + Precip1) was our top model when assessing survival for opportunistically captured neonates, and S(Canopy + Precip2) was our top model when assessing survival for all neonates regardless of capture type. Although we found variation in top models related to capture method, models including percent canopy cover and total precipitation during differing time intervals were competing in each candidate set albeit interpretation of total precipitation slightly varied among models (ranging from being unimportant to having a negative relationship with survival). However, variation in our results did not differ as drastically as they did for Gilbert et al. (2014); yet, our results still supported their conclusions and emphasize the importance of accounting for capture method in survival analyses when interpreting model selection results and effects of ecological covariates on survival.

We assumed results from our VIT‐only analysis best represented truth due to minimal left truncation in the dataset (Chitwood et al., 2017; Dion et al., 2020; Gilbert et al., 2014) and, therefore, only interpret those results relative to ungulate ecology. Our top model for 3‐month survival from our VIT‐only data supported our prediction that survival would vary by age and indicated survival was lowest early in life and increased later in life. Additionally, survival varying by three age intervals supported findings of Grovenburg et al. (2011) and Rohm et al. (2007) who noted that white‐tailed deer neonate survival varied by three age intervals with survival being lowest early in life and subsequently increasing with increased age. Our results only partially support Nelson and Woolf (1987) who found neonate survival varied by three age intervals; however, they reported survival was least during the second interval (i.e., 2 – 8 weeks of age). Nelson and Woolf (1987) attributed lower survival in the second interval to this age coinciding with white‐tailed deer neonates being mobile but not yet able to evade predators. Although variation in the results reported by Grovenburg et al. (2011), Rohm et al. (2007), and Nelson and Woolf (1987) may be related to how opportunistically caught neonates were aged (Grovenburg et al., 2014), our results better serve as a base for comparison as neonates included in our VIT‐only analysis were closest to known age. Ecological covariates affecting survival may also vary throughout the first 90 days of a neonate's life. For example, birth mass (Cook et al., 2004; Lomas & Bender, 2007; Shuman et al., 2017), sex (Shuman et al., 2017; Warbington et al., 2017), birth date (Plard et al., 2015; Michel, Gullikson, et al., 2020), and maternal age (Dion et al., 2020) likely affect survival of ungulate neonates; however, results vary (Dion et al., 2020; Kautz et al., 2019; Post et al., 2003). Assessing how these ecological covariates may influence neonate survival at specific age intervals (e.g., <2‐weeks, >2‐weeks) will allow for a better understanding of what affects neonatal ungulate survival throughout early life.

We also observed our S(Canopy + Precip1) survival model as competing for 3‐month survival from our VIT‐only dataset. Total amount of precipitation from 0 to 2 weeks of a neonate's life did not affect its survival. However, we identified a weak but positive relationship between neonate survival and percent canopy cover. Percent canopy cover may be an important feature on prairie landscapes due to the limited occurrence of forested cover, which comprised ≤ 9% of all cover types in our study. Additionally, although forested cover only comprised a small percentage of cover types in our study relative to grasslands and croplands, it may provide an important feature in helping neonates seek refuge from precipitation events, which can lead to hypothermia and subsequent death in neonates (Grovenburg et al., 2010, 2012; Linnell et al., 1995; Warbington et al., 2017). Other cover types likely provide cover from precipitation and other weather events as neonates tend to select bed sites with an increased understory in grassland landscapes (Grovenburg et al., 2010; Michel, Strickland, et al., 2020). However, we could not attribute any mortalities directly to hypothermia, though we cannot discount the potential for hypothermic neonates being more susceptible to predation and subsequent cause of death was classified as predation instead of hypothermia or hypothermia‐related mortalities being classified as unknown mortality events. Consequently, adequate canopy cover is likely needed for neonates to shelter from inclement weather.

Left truncation affected derived survival estimates, model selection, and interpretation of ecological covariates for 3‐month survival. However, capture method did not affect our interpretation of 6‐month survival, as it was not the top nor a competing model for our 6‐month survival candidate set. This result further supported Gilbert et al. (2014) who found capture method no longer affected survival estimates beyond 30 days for black‐tailed deer juveniles and Grovenburg et al. (2014) who found that age no longer affected 120‐day survival estimates for white‐tailed deer and mule deer (*O. hemionus*) juveniles. This result is important to consider when designing studies assessing ungulate survival. For example, if research is designed to assess factors affecting survival early in life (<3 months), then a capture method that minimizes left truncation (VITs) should be used. However, if research is designed to estimate factors affecting survival later in life (>3 months), then opportunistic capture methods are suitable.

Our top model describing 6‐month survival was S(Canopy + Precip2), which supported our prediction that percent canopy cover would positively affect survival while total precipitation would negatively affect juvenile survival. White‐tailed deer juveniles can be susceptible to hypothermia (Grovenburg et al., 2010, 2012; Linnell et al., 1995), and therefore, increased precipitation likely predisposes individuals to succumbing to hypothermia when adequate cover is unavailable (Warbington et al., 2017). Percent canopy cover likely provides the necessary cover to help juveniles thermoregulate during precipitation events. However, our results contradict those of Michel et al. (2018) who found that juvenile survival in the Northern Great Plains was related positively to total monthly precipitation. Differences in the effects of precipitation between our studies are likely related to scale as our study was comprised of 3 study areas in relatively close proximity, whereas Michel et al. (2018) conducted a meta‐analysis including 8 study sites across 3 states. Therefore, total precipitation during the parturition season likely has a negative impact on juvenile survival at local scales, whereas it has a positive impact on survival at large scales, potentially because of the relationship among total precipitation, quality of forage available to mothers, and maternal body condition (Michel et al., 2018). Consequently, understanding and interpreting variation in survival analyses relative to scale is important.

S(Year) was also a competing model when describing 6‐month survival indicating that cohort‐level effects persist until at least 6 months of age. We observed a 26% increased survival up to 6 months for neonates born in 2015 compared with those born in 2014. This suggests that environmental factors experienced in the year an individual was born may ultimately affect the number of individuals that survive and subsequently are recruited into a population. Cohort effects have been widely documented and can affect current year survival, current year reproductive success, future reproductive success, and individual phenotype (Douhard et al., 2013; Gaillard et al., 2003; Pigeon et al., 2017). Therefore, understanding cohort effects is not only necessary when assessing survival and reproduction, but also necessary for evaluating long‐term temporal trends in population fluctuations.

## 5 CONCLUSION

Our results show that survival rates vary by capture method up to 3 months of age for white‐tailed deer neonates. Additionally, even though there was some consistency among competing models among candidate sets analyzed by capture method (i.e., VIT only, opportunistic, and combined VIT and opportunistic captures), top models differed. Our interpretation of ecological covariates also differed among models, albeit interpretation did not vary as drastically as for Gilbert et al. (2014). Regardless, our results suggest that mortality varies by three time intervals, and therefore, models aimed to describe the relationship between ecological covariates and survival should be assessed by these time periods, particularly for models describing survival < 2 weeks of age. Survival rates derived from neonate white‐tailed deer captured opportunistically should also be adjusted downward by ~ 10%–25%. The presence of cohort effects also persisted up to 6 months indicating that conditions experienced during gestation and/or at birth have prolonged effects on survival. Finally, although forest is limiting in a prairie landscape, percent canopy cover is important as it likely provides cover from inclement precipitation events, which can negatively affect neonate survival up to 6 months of age.

## CONFLICT OF INTEREST

The authors declare to have no competing interests.

## AUTHOR CONTRIBUTION

**Katherine L Brackel :** Conceptualization (equal); Formal analysis (equal); Writing‐review & editing (equal). **Eric S Michel:** Conceptualization (equal); Investigation (equal); Supervision (equal); Writing‐original draft (equal). **Bailey S Gullikson:** Investigation (equal); Writing‐review & editing (equal). **Jonathan A Jenks:** Conceptualization (equal); Funding acquisition (equal); Investigation (equal); Methodology (equal); Resources (equal); Supervision (equal); Writing‐review & editing (equal). **William F Jensen:** Conceptualization (equal); Investigation (equal); Methodology (equal); Project administration (equal); Resources (equal); Supervision (equal); Writing‐review & editing (equal).

## Data Availability

Dataset has been deposited in DRYAD (https://doi.org/10.5061/dryad.37pvmcvjz).
